# Extended endoscopic transtuberculum approach for purely supradiaphragmatic pediatric craniopharyngiomas: outcomes from a single-center cohort study

**DOI:** 10.1007/s10143-025-03923-1

**Published:** 2025-11-21

**Authors:** Khaled Elshazly, Mohamed AR AbdelFatah, Mohamed Elsayed Youssef, Hazem A. Mostafa, Abdallah Maher Salem, MG EL Mashad, Sameh Hefny

**Affiliations:** 1 Neurosurgery Department, Faculty of Medicine, Ain Shams University, Cairo, Egypt; 2 Professor of Neurosurgery, Ain Shams University, Cairo, Egypt

**Keywords:** Pediatric craniopharyngiomas, Extended endoscopic endonasal approach, Surgical outcomes, Hypopituitarism, Visual outcomes

## Abstract

The extended endoscopic transtuberculum approach (EETA) for resecting purely supradiaphragmatic pediatric craniopharyngiomas lacks robust outcome data. This study evaluated the safety and efficacy of EETA in this population. A retrospective cohort study was conducted on consecutive pediatric patients who underwent EETA for purely supradiaphragmatic craniopharyngiomas (January 2018–July 2024) at a single institution. Outcomes included the extent of resection, complications, endocrine and visual function, adjuvant therapies, and recurrence. Among 14 patients (mean age 11.1 years, range 5–16), all presented with visual impairment, and 78.6% (*n* = 11) had preexisting hypopituitarism. The mean maximal tumor diameter was 3.6 cm (range 2.7–5.1), with hypothalamic involvement in 35.7% (*n* = 5). Gross total resection (GTR) was achieved in 5 cases (35.7%), and near-total resection (NTR) was achieved in 5 patients (35.7%). Postoperatively, 57.1% (*n* = 8) experienced visual improvement, while 14.3% (*n* = 2) worsened. Complications included cerebrospinal fluid leak with meningitis (7.1%, *n* = 1). New anterior pituitary deficits occurred in 35.7% (*n* = 5) and permanent diabetes insipidus developed in 85.7% (*n* = 12). New-onset obesity was observed in 28.6% (*n* = 4). At the 12-month follow-up, no tumor recurrence or progression was observed; residual tumors in 7 patients were managed with stereotactic radiosurgery. In this small cohort, the EETA was a viable surgical option for purely supradiaphragmatic pediatric craniopharyngiomas, offering favorable resection rates and visual outcomes in experienced hands. However, it carried significant endocrine risks. These preliminary findings require validation in larger, prospective studies with long-term follow-up.

## Introduction

Craniopharyngiomas are rare, histologically benign yet clinically aggressive tumors that present significant therapeutic challenges [1]. The standard transsphenoidal approach is well-established for resecting infradiaphragmatic craniopharyngiomas, which are located entirely or predominantly within an enlarged sella [2]. In contrast, the extended endoscopic transtuberculum approach (EETA) has emerged as a minimally invasive alternative to traditional transcranial routes for suprasellar lesions [3].

The EETA provides direct midline access to the suprasellar region without brain retraction, enabling enhanced visualization of the pituitary stalk and critical neurovascular structures. Additionally, it offers greater exposure of the subchiasmatic and retrochiasmatic spaces, facilitating safe microsurgical dissection of tumors from the undersurface of the optic chiasm and hypothalamus [4]. Despite these advantages, endoscopic approaches carry certain limitations, including higher rates of postoperative CSF leakage, reduced intraoperative hemorrhage control, and increased technical difficulty in accessing laterally extending tumors [5].

Furthermore, the EETA in pediatric patients presents unique challenges due to smaller nostrils, narrower nasal cavities, and reduced (or absent) sphenoid sinus pneumatization [6, 7]. Currently, the application of the EETA for purely supradiaphragmatic craniopharyngiomas in pediatric populations remains understudied, with limited data on surgical outcomes and complications. This single-center cohort study aims to evaluate the safety, efficacy, and clinical outcomes of this approach.

## Materials and methods

### Study design and population

We conducted a retrospective cohort study of all pediatric patients (< 18 years) who underwent an extended endoscopic endonasal transtuberculum approach (EETA) for a purely supradiaphragmatic craniopharyngioma at our institution between January 2018 and July 2024. Inclusion required a minimum postoperative follow-up of 12 months. The study cohort was identified from a database of 251 endoscopic endonasal procedures performed during this period.

### Ethical considerations

The Institutional Review Board of our Faculty of Medicine approved the study protocol (FWA 000017585). The research adhered to the ethical principles of the Declaration of Helsinki. Written informed consent for both the surgical procedure and the anonymous use of medical data was obtained from all parents or legal guardians.

### Reporting guidelines

This study was designed and reported in accordance with the STROBE (Strengthening the Reporting of Observational Studies in Epidemiology) guidelines for observational cohort studies.

### Data collection and variables

Patient medical records were systematically reviewed. Collected data included: demographic characteristics, history of previous interventions, preoperative assessment, operative details, postoperative care and complications, tumor histopathology, radiological findings, and clinical outcomes.

### Hormonal, ophthalmological, and obesity assessment

A complete pituitary hormonal profile was obtained preoperatively, within the first postoperative week, every 3 months during the first year, and annually thereafter. All patients underwent comprehensive preoperative and postoperative visual examinations. The body mass index standard deviation score (BMI SDS) was recorded preoperatively and at 3-month intervals; obesity was defined as a BMI SDS ≥ 2.

### Radiological assessment

Preoperative magnetic resonance imaging (MRI) with contrast was performed for all patients and repeated within 72 h postoperatively. Subsequent imaging was then performed at 6 and 12 months, and annually thereafter. Tumor size (defined as the longest diameter), morphology, location, and relationship to neurovascular structures were evaluated. Hypothalamic involvement was classified per Puget et al. (2007): Grade 0 (no involvement), Grade 1 (displacement), or Grade 2 (invasion) [8]. Preoperative head computed tomography (CT) assessed tumor calcification and sphenoid sinus pneumatization.

### Surgical technique and philosophy

A binostril, four-handed technique was employed. Our surgical philosophy was to achieve a maximal, yet safe, hypothalamic-sparing resection. The Puget classification was used preoperatively to stratify the risk of hypothalamic injury. The primary goal was hypothalamic preservation, even if it required leaving an adherent tumor capsule.

A right middle turbinectomy was performed. Neuronavigation guided a complete sphenoidotomy and wide drilling of the tuberculum sellae. The dura was opened above and below the superior intercavernous sinus, which was controlled with electrocautery. Following tumor capsule exposure, aggressive central debulking facilitated capsular mobilization. The pituitary stalk was identified and preserved whenever feasible. Careful direct inspection of the undersurface of the hypothalamus was performed; in cases of tumor invasion without a dissectible plane, the adherent capsule was left in situ to avoid hypothalamic injury.

### Reconstruction

The skull base defect was reconstructed with an extended fat graft, an onlay fascia lata graft, covered by a vascularized nasoseptal flap and fibrin sealant. A postoperative lumbar drain was maintained for 5 days at 10 mL/hour.

### Outcome assessment

The extent of resection was determined by the first postoperative MRI and classified as: gross total resection (GTR; no residual), near-total resection (NTR; >90% removed), subtotal resection (STR; 50–90% removed), and partial resection (PR) when < 50% of the tumor was removed. Tumor recurrence was defined as new growth after documented GTR. Tumor progression was defined as the growth of known residual tissue after NTR, STR, or PR. Primary outcomes included visual function, endocrinological status, and complications.

### Statistical analysis

Data were analyzed using IBM SPSS Statistics (Version 27). Continuous variables are presented as mean and range; categorical variables as numbers and frequencies.

## Results

### Patient demographics and clinical presentation

Fourteen consecutive pediatric patients met the inclusion criteria. Demographic characteristics and preoperative clinical features are summarized in Table 1. The most common presenting symptom was visual deterioration, occurring in all 14 patients (100%). Preoperative hydrocephalus, necessitating ventriculoperitoneal shunt (VPS) insertion, was present in 4 patients (28.6%). Three patients (21.4%) had a history of previous tumor resection at an external institution.

**Table 1:** Patient demographics and preoperative clinical characteristics

Characteristics	*n *(%)
Mean Age in years (Range)	11.1 (5-16)
Gender Male Female	9 (64.3) 5 (35.7)
Visual impairment	14 (100)
Headache	10 (71.4)
Endocrine Status	
Normal	3 (21.4)
Partial hypopituitarism	6 (42.9)
Panhypopituitarism	5 (35.7)
Hydrocephalus	4 (28.6)
Previous tumor surgery	
Transcranial	2 (14.3)
Ommaya Reservoir placement	1 (7.1)
Previous radiation	2 (14.3)

### Tumor characteristics

The tumor characteristics are detailed in Table 2. Histopathological examination confirmed adamantinomatous craniopharyngioma in all cases.

**Table 2:** Tumor characteristics and sphenoid sinus pneumatization

Characteristics	*N* (%)
Tumor size: Mean (range)	3.6 cm (2.7-5.1)
Consistency	
Cystic	1 (7.1)
Solid	2 (14.3)
Mixed (cystic and solid)	11 (78.6)
Tumor calcifications	9 (64.3)
Hypothalamic involvement	
Grade 0	2 (14.3)
Grade 1	7 (50)
Grade 2	5 (35.7)
Sphenoid Sinus Pneumatization	
Presellar	5 (35.7)
Sellar	6 (42.9)
Postsellar	1 (7.1)
Conchal	2 (14.3)

### Extent of resection and surgical outcomes

The extent of tumor resection is presented in Table 3. Gross total resection (GTR) was achieved in 5 cases (35.7%), and near-total resection (NTR) was achieved in 5 patients (35.7%). Consistent with our hypothalamic-sparing philosophy, no patients with Puget Grade 2 tumors underwent GTR. All patients with Puget Grade 2 tumors underwent either NTR (*n* = 2) or STR (*n* = 3), whereas GTR was achieved in 5 of the 9 patients with Puget Grade 0 or 1 tumors. The pituitary stalk was successfully preserved in 4 cases (28.6%).

**Table 3:** Outcomes and complications

Outcomes and Complications	*n* (%)
Extent of Resection	
Gross Total Resection	5 (35.7)
Near-Total Resection	5 (35.7)
Subtotal Resection	4 (28.6)
Visual outcome	
Improved	8 (57.1)
Stable	4 (28.6)
Worsened	2 (14.3)
Endocrine Outcomes	
Permanent Diabetes Insipidus	12 (85.7)
New Anterior Pituitary Deficit	5 (35.7)
Permanent Panhypopituitarism	10 (71.4)
Hormonal Recovery	0 (0)
Postoperative CSF Leak	1 (7.1)
Meningitis	1 (7.1)
Shunt malfunction/Hydrocephalus	2 (14.3)
New-Onset Obesity	4 (28.6)
Major vessel injury/Stroke	0 (0)
Mortality	0 (0)

### Postoperative complications and clinical outcomes

Postoperative complications and outcomes are summarized in Table 3. Visual improvement was observed in 8 cases (57.1%). Postoperative visual deterioration occurred in 2 patients (14.3%). Among the 4 patients with pituitary stalk preservation, 2 (50%) still developed permanent diabetes insipidus (DI). No patient with preoperative partial or panhypopituitarism experienced hormonal recovery. Five patients (35.7%) developed new, permanent anterior pituitary deficits.

One patient (7.1%) developed a postoperative CSF leak, which was successfully managed with endoscopic revision; this patient subsequently developed meningitis, which resolved with intravenous antibiotics. Two patients (14.3%) experienced VPS malfunction requiring revision. There were no vascular injuries, strokes, or deaths.

### Follow-up and oncological outcomes

The mean follow-up duration was 28.3 months (range: 12–45 months). Postoperative stereotactic radiosurgery (SRS) was offered to all patients with residual tumors, except for two with remnants adherent to the optic chiasm. This included all 4 patients with STR and 3 of the 5 with NTR. The other 2 NTR patients had a thin, calcified remnant adherent to the optic chiasm, for which SRS was deemed unsafe; their parents opted for observation. At the 12-month follow-up, no tumor recurrence or progression was observed.

## Discussion

This study presents a homogeneous cohort of pediatric patients with purely supradiaphragmatic craniopharyngiomas treated via EETA. Our findings suggest that this approach can be a viable surgical option in specialized centers, though it is associated with significant endocrine morbidity. A primary objective was achieving maximal safe resection. In our cohort, GTR was achieved in 35.7% of cases and NTR in another 35.7%. Direct comparisons with other studies are challenging, as most series combine sellar, suprasellar, and transdiaphragmatic tumors, which inherently have different resection profiles. Our results appear consistent with the literature when contextualized; for instance, Caklili et al. (2023) reported higher GTR rates in a mixed cohort, noting that rates were highest for purely sellar lesions [9]. A summary of selected literature is presented in Table 4 [10–13], which places our findings in a broader context.

**Table 4:** Summary of selected literature on endoscopic endonasal surgery for pediatric craniopharyngiomas with supradiaphragmatic components

Study	n	Tumor location	Gross total resection	Visual improvement	CSF leak*n* (%)	New onset hormone deficit*n* (%)	New onset DI	Newonset obesity	Meanfollow-up	recurrence/ progression
Alalade et al.2017 (10)	11	5 Suprasellar	45%	73%	1	81.8%	63.3%	2(18%)	36 months for 60% of cases	1 (9%)
d’Avella et al.2019 (11)	12	7infradiaphragmatic5Supradiaphragmatic	75%(60% for the supradiaphragmatic group)	91%	One case(8)	3(60)	4(36%)	Not stated	78 months	40% for supradiaphragmatic
Scheliniet al.2019 (12)	20	Not identified	14(70%)	4/20 had vision affection before and did not change	1(5%)	55%	Notstated	Notstated	63.3 months	3(15%)
Javadpour et al.2021 (13)	15	Not identified	4(27%)	69.2%	0	54%	75%	1 (7%)	74 months	2 (13%)
OUR STUDY	14	AllSupradiaphragmatic	35.7% GTR	57%	1(7.1%)	5 (35.7%)	12(85.7%)	428.6%	28.3 months	None in one year

The significant challenges and steep learning curve associated with the pediatric EETA must be emphasized. We attribute the technical outcomes in this small cohort to our team’s cumulative experience, with each member having performed over 300 endoscopic endonasal procedures in both adult and pediatric patients, providing the extensive experience necessary to manage the unique anatomical constraints in children effectively. Navigating smaller nasal passages was achieved by leveraging the shorter skull base distance and utilizing a binostril, four-handed technique aided by neuronavigation.

Our surgical philosophy centered on a hypothalamic-sparing approach. For any Puget grade 2 hypothalamic invasion, we exercised extreme caution, intentionally leaving adherent tumor capsules in situ based on preoperative imaging and direct intraoperative inspection to avoid injury.

The management of residual disease is an important consideration. Patients with residual tumors were referred for consideration of SRS or fractionated stereotactic radiotherapy (FSRT). We acknowledge that FSRT is the more established standard for complex residuals, and a longer follow-up is necessary to validate the efficacy and safety of the SRS protocol used in our patients.

Despite the resection rates, the approach carried a high risk of endocrinopathy. New anterior pituitary deficits occurred in 35.7%, and 85.7% developed permanent DI. Notably, even among the four patients in whom the pituitary stalk was anatomically preserved, half still developed permanent DI. These findings are consistent with other reports [14], underscoring the vulnerability of the hypothalamic-pituitary axis.

Visual outcomes were largely positive, with improvement in 57.1% of cases, aligning with other series [15]. Our major complication rate was low, with a 7.1% CSF leak rate, comparable to published rates [16, 17], which we attribute to a meticulous multilayer reconstruction technique. Hypothalamic morbidity, observed as new-onset obesity in 28.6% of our cohort, remains a critical concern.

### Study limitations

The principal limitations are the retrospective design, small sample size, and single-institution origin, which may affect generalizability. The analysis is constrained by a relatively short-term follow-up and the absence of structured neurocognitive and quality-of-life data. Despite these limitations, this study provides a unique and homogeneous dataset for this specific tumor subgroup.

## Conclusion

In this small, single-center cohort, the EETA was a viable and effective surgical option for resecting purely supradiaphragmatic pediatric craniopharyngiomas, offering favorable rates of resection and visual improvement with an acceptably low rate of major surgical complications when performed by an experienced team. However, the approach was associated with significant endocrine morbidity, emphasizing the need for meticulous technique and comprehensive preoperative counseling. These preliminary findings require validation through future prospective, multi-institutional studies with larger cohorts and longer follow-up.

## Data Availability

No datasets were generated or analysed during the current study.
